# Forecasting multidrug-resistant organisms infection trends in a Chinese tertiary hospital (2014–2024): a comparative study of SARIMA, ETS, Prophet, and NNETAR models

**DOI:** 10.3389/fpubh.2026.1687658

**Published:** 2026-01-29

**Authors:** Haiyan Chen, Luojing Zhou

**Affiliations:** 1 School of Basic Medical Sciences and School of Public Health, Faculty of Medicine, Yangzhou University, Yangzhou, Jiangsu, China; 2 Northern Jiangsu People’s Hospital, Yangzhou, Jiangsu, China

**Keywords:** ETS, forecasting, infection control, multidrug-resistance, NNETAR, Prophet, SARIMA

## Abstract

**Background:**

Infections caused by multidrug-resistant organisms (MDROs) continue to pose serious challenges for hospital infection control, often resulting in longer hospitalizations, increased patient morbidity, and higher healthcare costs. While time series forecasting has gained traction as a tool for anticipating MDROs trends, there remains a lack of real-world studies comparing the effectiveness of different modeling approaches using hospital-based data.

**Objective:**

This study aimed to evaluate and compare the predictive performance of four time series models—SARIMA, ETS, Prophet, and NNETAR—using monthly MDROs infection data collected from a tertiary hospital in China between 2014 and 2023, with the goal of forecasting trends for 2024.

**Methods:**

Monthly MDROs infection rates from January 2014 to December 2023 were analyzed using R software. Stationarity was assessed through unit root tests, and appropriate differencing was applied as needed. Each model was fitted to the training dataset and used to forecast infection rates for the year 2024. Model accuracy was assessed by comparing forecasted values with actual 2024 data using root mean squared error (RMSE), mean absolute error (MAE), mean absolute percentage error (MAPE), symmetric mean absolute percentage error (sMAPE), and mean absolute scaled error (MASE).

**Results:**

Among the models, SARIMA produced the most consistent and reliable forecasts (RMSE = 0.0469, MAE = 0.0424, MAPE = 20.74%, sMAPE = 21.27%, MASE = 0.932), with residuals satisfying tests for independence and normality. Although the ETS model achieved lower numerical point errors (RMSE = 0.0367, MAE = 0.0305, MAPE = 14.46%, sMAPE = 14.81%, MASE = 0.670), its residual diagnostics raised concerns regarding robustness. The Prophet (RMSE = 0.0499, MAE = 0.0439, MAPE = 20.41%, sMAPE = 22.15%, MASE = 0.563) and NNETAR (RMSE = 0.0697, MAPE = 30.60%, sMAPE = 30.60%, MASE = 0.072) models captured certain aspects of the data dynamics but showed lower overall robustness compared with SARIMA.

**Conclusion:**

Based on its overall robustness and diagnostic consistency, SARIMA is recommended for short- to medium-term forecasting of MDROs infection trends. The other models, while less reliable on their own, may still be valuable for validating trends and conducting sensitivity analyses to support hospital infection control planning.

## 1 Introduction

Multidrug-resistant organisms (MDROs) have become a major challenge to global public health, particularly in hospital environments where high antibiotic exposure and weakened patient immunity increase the risk of transmission (1, 2). In recent years, the incidence of MDROs-related infections has risen markedly, leading to higher rates of morbidity, extended hospitalization periods, and greater medical expenses (3). As a result, accurate forecasting of MDROs infection trends plays a critical role in healthcare planning and the timely implementation of preventive measures (4).

Among widely used time series methods, the SARIMA model has been a frequent choice in infectious disease forecasting. Compared to the more basic ARIMA model, SARIMA is better suited to reflect seasonal changes that often appear in epidemiological data (5). In addition to SARIMA, other models—such as the Exponential Smoothing State Space (ETS) model (6), Prophet developed by Facebook (7), and Neural Network Autoregression (NNETAR) (8)—have recently drawn attention for their ability to handle nonlinear patterns, long-term shifts, and seasonality.

Although a variety of forecasting models are now available, only a limited number of studies have directly compared their predictive performance in monitoring MDROs infections within hospital settings. In addition, their forecasting accuracy and residual behaviors have not been fully assessed using up-to-date clinical data.

In this study, we analyzed monthly MDROs infection rates reported by a tertiary hospital in China from January 2014 to December 2023. Four time series forecasting models—SARIMA, ETS, Prophet, and NNETAR—were developed and compared to estimate infection trends for the year 2024 and to determine which model offered the most reliable performance. Their accuracy was assessed using standard forecasting metrics (RMSE, MAE, MAPE, sMAPE, MASE) (9) along with residual analysis, with the goal of supporting data-informed strategies for infection control.

## 2 Methods

### 2.1 Data collection and preprocessing

We collected monthly data on infection rates of multidrug-resistant organisms (MDROs) from a tertiary hospital in China, spanning January 2014 to December 2023. The dataset was anonymized and aggregated on a monthly basis. Infection rate was calculated as the number of MDROs infection cases divided by the total patient-days per month and expressed as a percentage.

Data preprocessing was conducted using R (version 4.1.2), including standardizing date formats and addressing missing or irregular values. The final series was analyzed as a univariate time series (10) for model development.

### 2.2 Model construction

#### 2.2.1 SARIMA model

The Seasonal Autoregressive Integrated Moving Average (SARIMA) model is denoted as SARIMA(p,d,q)(P,D,Q)s (11), where p, d, and q represent the orders of the non-seasonal autoregressive, differencing, and moving average components, respectively (12). P, D, and Q refer to the seasonal counterparts of these parameters, and s indicates the length of the seasonal cycle (e.g., s = 12 for monthly data with yearly seasonality).

The modeling process involves the following steps:

Stationarity transformation:

To assess whether the time series is stationary, the Augmented Dickey–Fuller (ADF) test is commonly applied (13). When non-stationarity is detected, the data are typically transformed through methods such as differencing, seasonal differencing, or logarithmic transformation to stabilize both the mean and variance.

Model identification and parameter estimation:

Initial model orders are typically identified by examining autocorrelation function (ACF) and partial autocorrelation function (PACF) plots (14, 15). In addition, a stepwise search procedure may be used, in which various parameter combinations within a defined range are tested, and the model yielding the lowest Akaike Information Criterion (AIC) is selected.

Diagnostic checking:

The adequacy of the fitted model is assessed by analyzing residual plots and applying the Ljung–Box test (16). If the test yields a non-significant *p*-value (*p* > 0.05), the residuals can be considered approximately white noise, suggesting that the model provides a good fit to the data.

Forecasting:

The final selected model is used to generate forecasts along with prediction intervals for future time points.

#### 2.2.2 NNETAR model

The Neural Network Autoregression (NNETAR) model is a nonlinear and nonparametric method for time series forecasting that uses artificial neural networks. The modeling process involves two main steps. First, an autoregressive (AR) model (17) is fitted to the data to determine the optimal number of non-seasonal lags (p), based on the lowest Akaike Information Criterion (AIC). Then, the selected p non-seasonal lags and P seasonal lags are used as inputs to a feed-forward neural network, with the actual observed value as the output. The model includes k hidden neurons and is commonly written as NNETAR (p,P,k)m, where m is the seasonal period (for example, m = 12 for monthly data).

This model can capture nonlinear relationships and complex seasonal patterns, which makes it a good choice for time series that are challenging to model with traditional linear methods.

#### 2.2.3 ETS model

The ETS (Error-Trend-Seasonality) model is a family of time series forecasting methods built on exponential smoothing techniques. It is commonly written as ETS(Z,Z,Z), where the three letters indicate the types of error, trend, and seasonality components. Each element can be specified as additive (A), multiplicative (M), or none (N), depending on the characteristics of the data.

Model parameters are estimated using Maximum Likelihood Estimation (MLE) (18). Different combinations of error, trend, and seasonality components are tested to fit various model structures, and the one with the lowest Akaike Information Criterion (AIC) is chosen. ETS models work well for time series that show clear trends or seasonality and are flexible enough to capture structural changes over time.

#### 2.2.4 Prophet model

The Prophet model, developed by Facebook, is well-suited for time series data that exhibit nonlinear trends and seasonal patterns. It decomposes a time series into three components—trend, seasonality, and holidays—which are modeled independently and combined in an additive manner.

Prophet is designed for flexibility and robustness, enabling it to handle missing data, outliers, and complex seasonal patterns effectively. Compared with traditional ARIMA models, it requires fewer assumptions and minimal manual adjustment, making it a practical option for forecasting applications in healthcare and public health.

The model supports piecewise linear growth trends and explicitly incorporates seasonal effects within a Bayesian framework. In this study, the Prophet model was implemented using its default configuration, with yearly seasonality enabled and without the inclusion of holiday effects or additional regressors.

### 2.3 Forecasting and model evaluation

For the SARIMA model, the optimal set of parameters was selected based on the Akaike Information Criterion (AIC) and the Bayesian Information Criterion (BIC) (19). The specification with the lowest AIC and BIC values was regarded as the best-fitting model.

To assess the predictive performance of each model, we applied three commonly used error metrics: root mean squared error (RMSE), mean absolute percentage error (MAPE), and mean absolute error (MAE), symmetric mean absolute percentage error (sMAPE), and mean absolute scaled error (MASE).

### 2.4 Data processing and analysis

All analyses were conducted using R software (version 4.1.2; R Foundation for Statistical Computing, Vienna, Austria). A significance level of 0.05 was used for all statistical tests.

## 3 Results

### 3.1 Overall trend of infection rates

Between 2014 and 2024, the monthly multidrug-resistant organisms (MDROs) infection rates in the hospital exhibited a fluctuating but generally declining trend. During the early years (2014–2016), the infection rates showed high-frequency oscillations, mostly ranging between 0.25 and 0.40, with a local peak in 2015. From 2017 to 2019, the fluctuations narrowed slightly but remained moderate overall.

In early 2020, the infection rate surged rapidly, reaching its maximum value in February (exceeding 0.6), then sharply declined. This spike may be attributed to changes in healthcare-seeking behaviors and resource allocation during the early stage of the COVID-19 outbreak (20).

**Figure 1:**
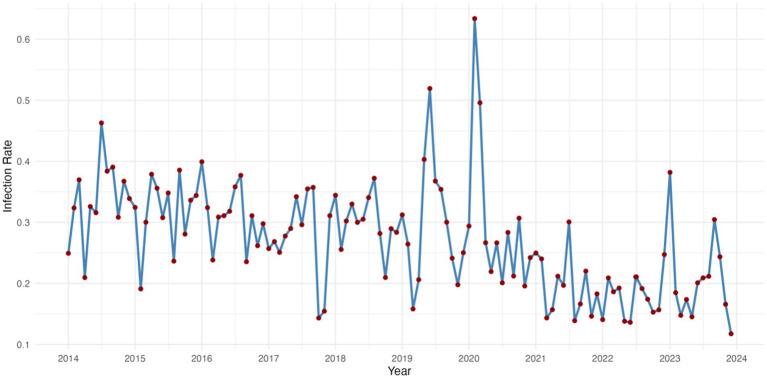
Time series of infection rates for multi-drug resistant bacteria 2014–2023.

During 2021–2022, the infection rates significantly decreased and remained relatively stable, mostly below 0.20. A slight rebound was observed in 2023, peaking in September (around 0.38), followed by another decline. In 2024, the rates have remained low, ranging from 0.14 to 0.26, suggesting some effectiveness in hospital infection control while seasonal variation and potential rebound still warrant close attention. As shown in Figure 1.

### 3.2 Time series decomposition analysis

**Figure 2:**
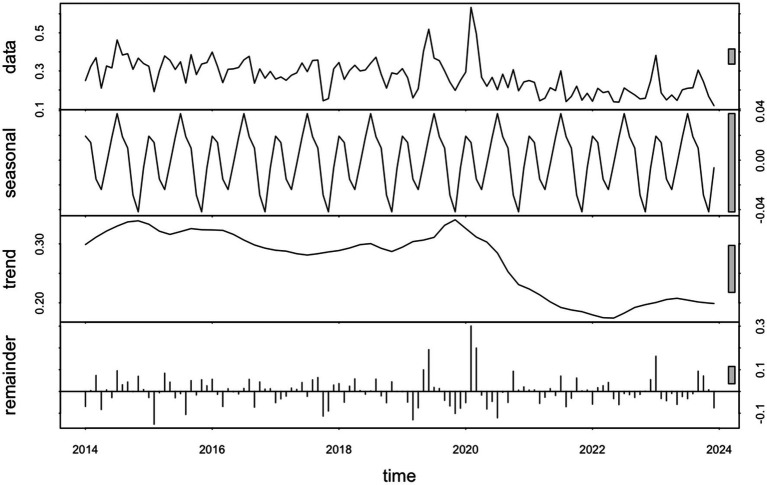
STL decomposition of monthly infection rates.

To better understand the structure of the infection rate time series, we performed a Seasonal-Trend decomposition using Loess (STL) (Figure 2). The decomposition revealed a clear seasonal component with regular annual cycles, as well as a downward trend after peaking around 2019. The residual component showed relatively small fluctuations, indicating that most of the variation can be explained by trend and seasonality. Therefore, a SARIMA model is appropriate for capturing the temporal dependencies and forecasting future trends.

### 3.3 SARIMA model construction and diagnostics

**Figure 3:**
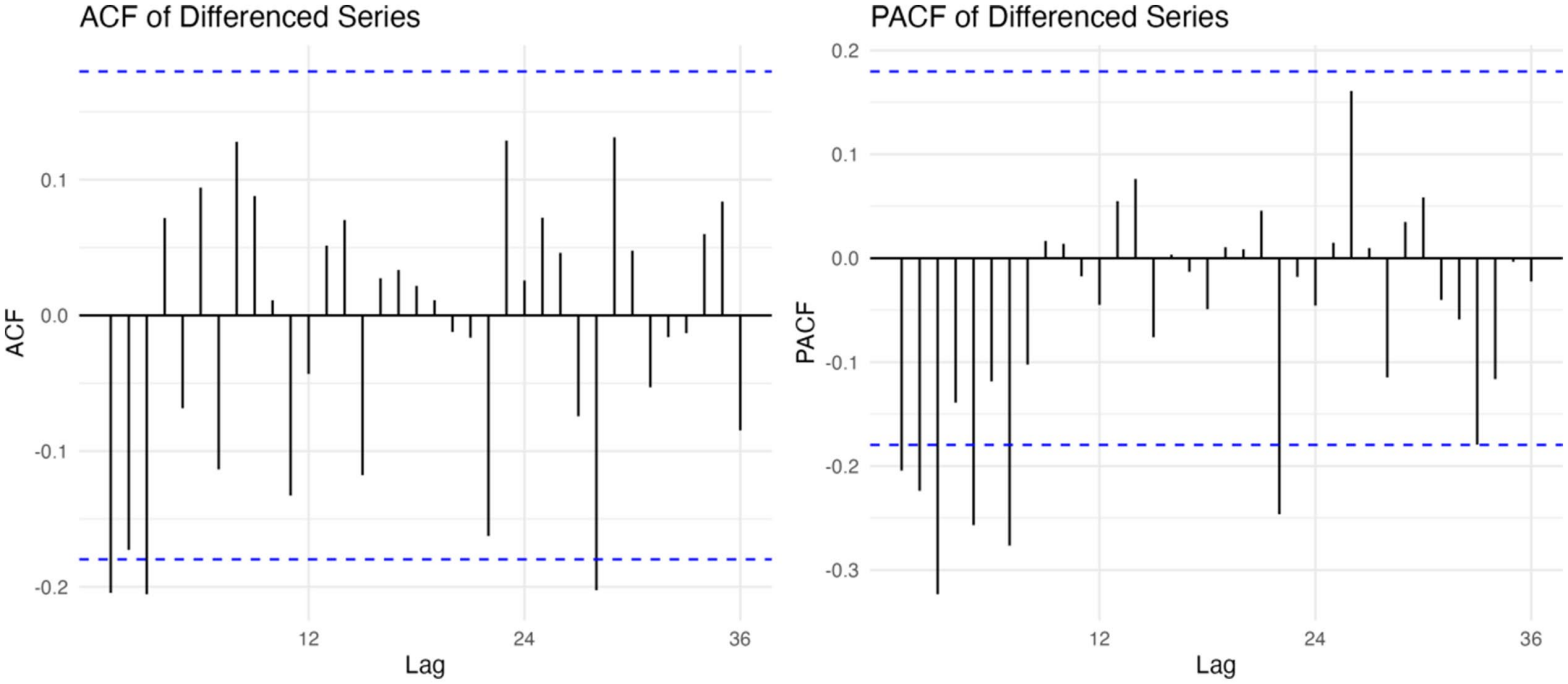
ACF and PACF plots of the first-order differenced series.

Before constructing the SARIMA model, the stationarity of the original time series was examined. The Augmented Dickey–Fuller (ADF) test statistic was −4.8576 (*p* < 0.01), suggesting stationarity. Nevertheless, the ndiffs() function recommended one order of differencing, and applying first-order differencing further improved model adequacy. The differenced series showed stronger evidence against a unit root (ADF = −8.6267, *p* < 0.01), lower information criteria (AIC = −223.35, BIC = −207.31), and residuals closer to white noise. Autocorrelation function (ACF) and partial autocorrelation function (PACF) plots of the differenced series (Figure 3), together with AIC/BIC minimization and a stepwise trial-and-error procedure, guided the selection of candidate models. The SARIMA(2,1,2)(0,1,1)[12] specification was ultimately chosen as the optimal model.

**Figure 4:**
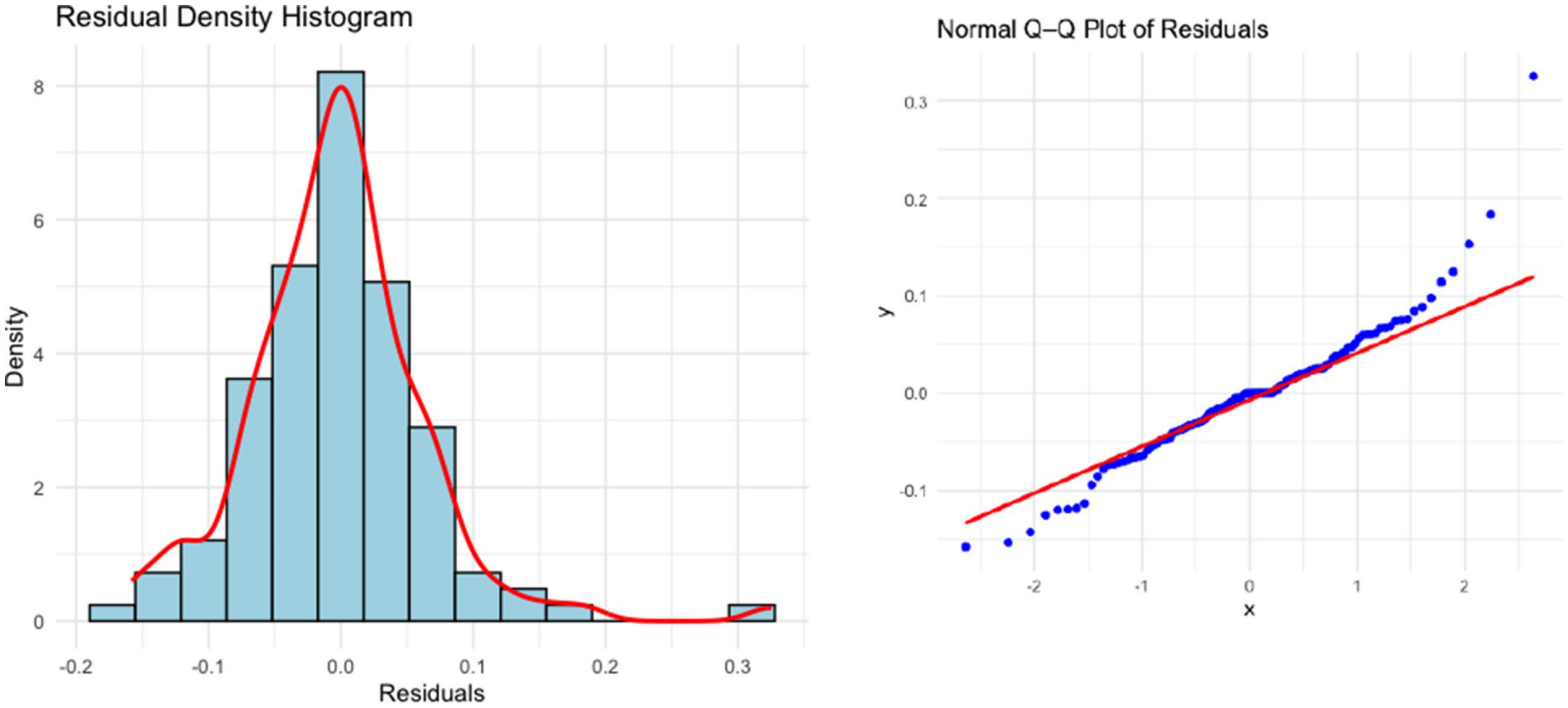
Density histogram and normal Q-Q diagram of the residuals in SARIMA(2,1,2)(0,1,1)[12] model.

The residuals of the model passed the Ljung-Box test (lag 20) with a *p*-value of 0.3225, indicating no significant autocorrelation. In addition, the residuals followed an approximate diagonal pattern in the normal Q-Q plot, and the Shapiro–Wilk test yielded (21) a *p*-value > 0.05, supporting the normality of the residuals. These results suggest that the model fits well and meets the conditions required for forecasting (see Figure 4).

**Figure 5:**
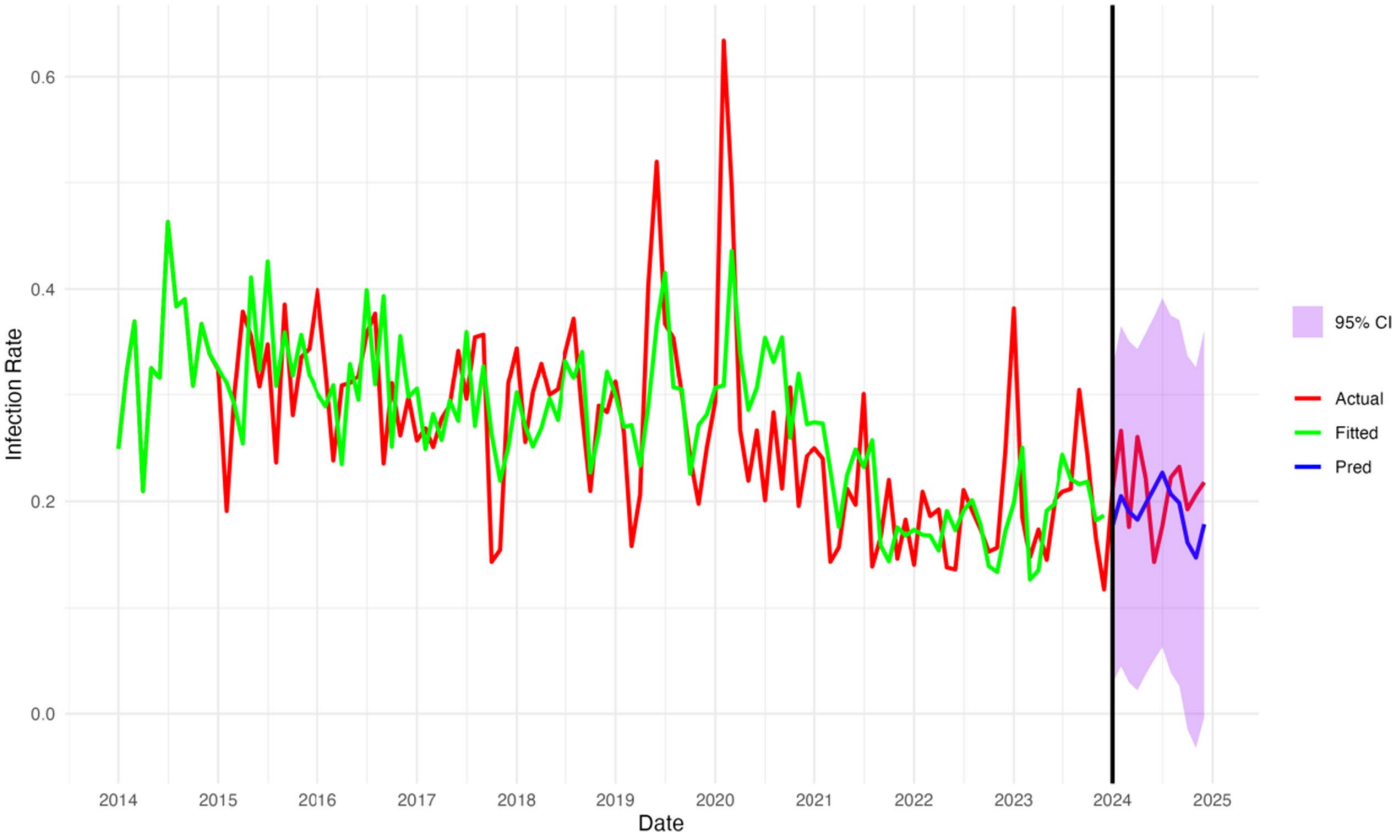
Fitted and forecasted monthly MDROs infection rates from 2014 to 2024 using the SARIMA model.

As shown in Figure 5, the SARIMA model demonstrates excellent fitting and forecasting performance for multidrug-resistant organisms (MDROs) infection rates. During the training period (2014–2023), the fitted values closely follow the trend of the actual values, indicating high fitting accuracy. In the forecasting period (2024), the predicted curve remains stable, and most predicted values fall within the reasonable confidence interval, suggesting the model has strong trend-capturing ability and forecasting stability. Overall, the SARIMA model exhibits good performance in both fitting and prediction in this study.

### 3.4 Prophet model construction and diagnostics

**Figure 6:**
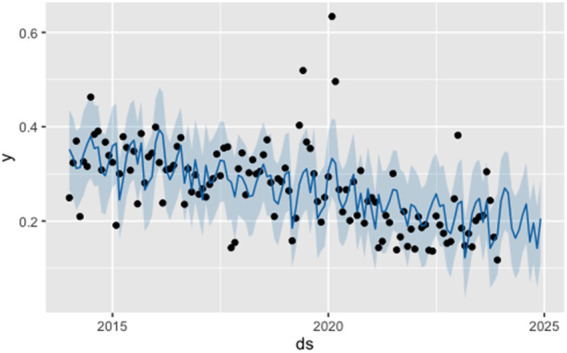
Forecast of infection rates from January to December 2024 using the Prophet model. Black dots represent observed values, the blue line indicates fitted or predicted values by the Prophet model, and the shaded area shows the 95% confidence interval.

In this study, the Facebook Prophet model was employed to model and forecast the same time series. The input data frame for the Prophet model consisted of two columns: the date (ds) and the infection rate (y), with monthly granularity. During model specification, yearly seasonality was enabled (yearly.seasonality = TRUE), while weekly and daily seasonalities were disabled. No holiday regressors were included. The model generated rolling forecasts for infection rates from January to December 2024; the prediction curve and 95% confidence intervals are shown in Figure 6.

**Figure 7:**
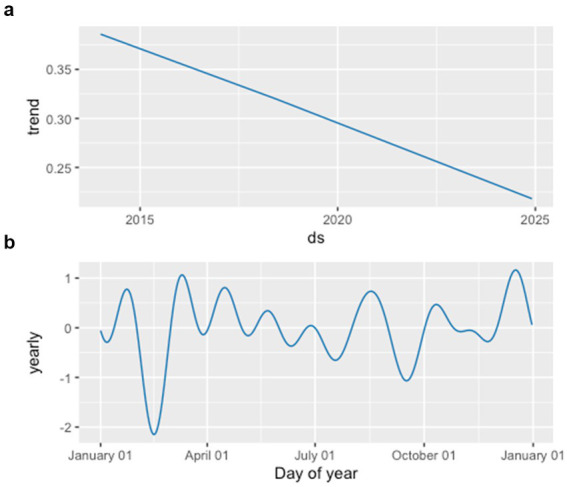
Decomposition of monthly infection rates of multidrug-resistant organisms: **(a)** effect of the overall trend and **(b)** effect of yearly seasonality.

The decomposition results (Figure 7) revealed that the infection rate series consists of a slowly declining long-term trend and a stable yearly seasonal pattern. The seasonal component exhibits relatively higher values during March–May and October–December, which aligns with the seasonal fluctuations identified by the SARIMA model.

### 3.5 ETS model construction and diagnostics

In this study, the ETS model was employed to fit the time series of infection rates. The optimal model automatically selected by R was ETS(A,N,N), indicating an additive error term with no trend or seasonal components. This suggests that the data exhibit relatively stable fluctuations. The model yielded an AIC value of −38.87, indicating a good fit. The ETS model was automatically selected using information criteria. Although seasonal patterns were suggested by STL decomposition, the inclusion of a seasonal component did not improve model fit according to AICc, and a non-seasonal ETS specification was therefore selected.

Residual diagnostics showed the following: The Ljung-Box test produced a statistic of Q = 46.98 (df = 24, *p* = 0.0034), suggesting that residuals exhibit some autocorrelation; The Shapiro–Wilk normality test returned W = 0.931 (*p* < 0.001), indicating that the residuals do not follow a normal distribution.

Therefore, although the ETS model achieved low point forecast errors, the presence of residual autocorrelation and non-normality limits its suitability as the primary forecasting model for routine epidemiological surveillance.

### 3.6 NNETAR model construction and diagnostics

This study employed the Neural Network Autoregression (NNETAR) model to forecast the monthly infection rate series. NNETAR is a non-parametric, nonlinear forecasting approach capable of capturing complex patterns that may not be adequately represented by linear time series models.

The model was implemented using the nnetar() function in R. Based on the training data, the lag orders and the number of hidden nodes were automatically selected, resulting in an NNAR(11,1,6)[12] specification.

Residual diagnostics indicated no significant autocorrelation (Ljung–Box Q = 13.01, *p* = 0.877), suggesting an adequate model fit. The Shapiro–Wilk test indicated deviation from normality (W = 0.779, *p* < 0.001). However, as neural network–based forecasting methods do not rely on distributional assumptions regarding residuals, this characteristic does not invalidate the model’s use for forecasting. Accordingly, the NNETAR model was included as a complementary and exploratory nonlinear forecasting approach.

### 3.7 Model evaluation

In this study, four time series forecasting models—SARIMA, ETS, Prophet, and NNETAR—were applied to predict the monthly MDROs infection rates in 2024. The models were evaluated using five error metrics: root mean squared error (RMSE), mean absolute error (MAE), mean absolute percentage error (MAPE), symmetric mean absolute percentage error (sMAPE), and mean absolute scaled error (MASE). The results are summarized as follows (Table 1): The SARIMA model demonstrated balanced performance across all metrics, with RMSE = 0.0469, MAE = 0.0424, and MAPE = 20.74%, sMAPE = 21.27%, MASE = 0.932. It showed strong predictive accuracy and stability, and passed residual diagnostics, making it the most reliable model in this study. The ETS model achieved the lowest prediction errors (RMSE = 0.0367, MAE = 0.0305, MAPE = 14.46%, sMAPE = 14.81%, MASE = 0.670); however, its residuals exhibited significant autocorrelation and deviation from normality, which raises concerns regarding robustness. It is not recommended as the primary forecasting model for routine surveillance.

**Table 1:** Model performance comparison based on forecast error metrics.

Model	RMSE	MAE	MAPE (%)	sMAPE (%)	MASE
SARIMA(2,1,2)(0,1,1)[12]	0.0469	0.0424	20.74	21.27	0.932
ETS(A,N,N)	0.0367	0.0305	14.46	14.81	0.670
Prophet	0.0499	0.0439	20.41	22.15	0.563
NNETAR(11,1,6)[12]	0.0697	0.0608	30.60	30.60	0.072

Thus, it is not recommended as the final forecasting model. The Prophet model effectively captured the trend and seasonal components of the data. While its prediction errors (RMSE = 0.0499, MAE = 0.0439, MAPE = 20.41%, sMAPE = 22.15%, MASE = 0.563) were slightly higher than those of SARIMA, the model still performed well and can serve as a valuable complementary approach. The NNETAR model attempted to capture nonlinear patterns, but its predictive accuracy was relatively poor (RMSE = 0.0697, MAE = 0.0608, MAPE = 30.60%, sMAPE = 30.60%, MASE = 0.072), suggesting that it may be more suitable for exploratory analysis rather than primary forecasting tasks. In summary, the SARIMA model demonstrated the best overall performance in terms of fitting accuracy, forecast reliability, and residual behavior, making it the most appropriate choice for predicting MDROs infection trends in this study. The other models can be used as supplemental tools or for sensitivity analyses.

## 4 Discussion

This study systematically compared the forecasting performance of four time series models—SARIMA, ETS, Prophet, and NNETAR—using MDROs infection rate data from a tertiary hospital in China between 2014 and 2023. Among these models, SARIMA demonstrated the most reliable overall performance when predictive accuracy, residual diagnostics, and interpretability were considered together, making it a reliable choice for public health forecasting.

The SARIMA model effectively captured both seasonal variations and long-term trends in the MDROs infection data through its structured seasonal components. In the 2024 forecast, it outperformed the other models, achieving an RMSE of 0.047, MAE of 0.042, and MAPE of 20.7%, sMAPE of 21.27%, MASE of 0.932. Residual diagnostics indicated no significant autocorrelation or deviation from normality, highlighting the model’s robustness and reliability. An evident peak was observed in early 2020, with the MDROs infection rate exceeding 0.6 in February as captured by the Prophet model. This abrupt increase coincided with the COVID-19 outbreak and was treated as a real-world disruption rather than a data artifact; therefore, the observation was retained without imputation or exclusion. Different forecasting models respond to abrupt changes in distinct ways. In particular, Prophet incorporates flexible trend components with automatic change-point detection, which may allow it to better adapt to sudden level shifts associated with large-scale disruptions such as the COVID-19 pandemic. In contrast, more rigid linear models, such as SARIMA and ETS, may smooth extreme fluctuations depending on their estimated structure. Nevertheless, while Prophet may be more responsive to structural changes, fully characterizing pandemic-related regime shifts may require dedicated intervention models or the inclusion of exogenous indicators reflecting healthcare disruption. In the post-pandemic period, MDROs trends may be influenced by evolving infection control practices, antibiotic stewardship, and changes in healthcare utilization, highlighting the need for continued temporal validation using post-2024 data.

Although the ETS model showed reasonable performance on the training data, its residuals displayed significant autocorrelation (Ljung–Box *p* = 0.003), violating the assumption of white noise. In addition, the absence of explicit trend and seasonality components limits its suitability for long-term epidemiological forecasting. The Prophet model, which is known for its flexible trend decomposition and success in business applications, performed slightly less accurately in this study (MAPE = 20.41%). This may be attributed to the exclusion of holiday effects or other external variables in the model configuration.

The NNETAR model offers the advantage of capturing nonlinear relationships and complex lag structures. Although the NNETAR model yielded a very low MASE value, this reflects comparison against a naïve benchmark rather than superior absolute forecast accuracy, as indicated by its higher RMSE and MAE.

However, its forecasting performance was less consistent, with a MAPE of 30.60% for the 2024 predictions. In addition, the residuals did not meet the assumption of normality (W = 0.779, *p* < 0.001). These limitations may be due to the model’s sensitivity to training data, a tendency to overfit, and weaker seasonal pattern handling compared to the SARIMA model.

This study underscores the importance of accurate forecasting models in guiding infection control efforts and optimizing resource allocation. The SARIMA model, with its strong predictive performance, can serve as an early warning tool that enables hospitals to respond proactively to emerging infection trends, helping to reduce MDROs transmission and support more effective antibiotic stewardship.

From an infection prevention perspective, the forecasts generated in this study are best interpreted as early warning signals rather than precise operational thresholds. For example, a sustained increase in the predicted MDROs infection rate may indicate heightened transmission risk and could prompt closer surveillance, reinforcement of patient cohorting, intensified environmental cleaning, or escalation of antimicrobial stewardship efforts.

Short-term forecasts, particularly at 1–3 month horizons, may offer practical value for anticipatory planning and timely intervention. Nevertheless, the translation of forecast outputs into explicit decision thresholds or utility-based strategies requires integration with local clinical context and was beyond the scope of the present analysis.

Despite the methodological rigor of this study, several limitations should be considered. First, as the dataset was obtained from a single tertiary hospital, the generalizability of the findings to other settings may be limited. Regional and institutional factors may influence MDROs infection trends through several mechanisms. For example, antibiotic stewardship policies in Jiangsu Province may affect prescribing practices and antimicrobial selection pressure, thereby shaping local resistance patterns. In addition, tertiary hospitals typically manage more complex and critically ill patients with higher device utilization, which may lead to different baseline MDROs rates and temporal dynamics compared with smaller hospitals. Therefore, the findings should be interpreted within this specific institutional and regional context.

Second, the forecasting models did not include exogenous factors such as antibiotic consumption, ward-level epidemiological changes, or holiday influences, all of which could impact prediction accuracy. Finally, this study focused on point forecast accuracy, and a systematic comparison of prediction interval coverage, width, and calibration across models was beyond the scope of the current analysis.

Future studies may benefit from integrating multi-source data—such as electronic medical records and antibiotic administration logs—into multivariate forecasting models to enhance predictive accuracy. In addition, validating the models with multicenter datasets and exploring hybrid approaches that combine traditional time series methods with machine learning or deep learning techniques could further improve their generalizability and practical utility.

## 5 Conclusion

This study constructed and compared four time series forecasting models—SARIMA, ETS, Prophet, and NNETAR—based on monthly multidrug-resistant organisms (MDROs) infection rates from a tertiary hospital in China spanning 2014 to 2023. The goal was to evaluate their predictive performance and potential for hospital infection surveillance.

The results demonstrated that the SARIMA(2,1,2)(0,1,1)[12] model demonstrated the most reliable overall performance when predictive accuracy, residual behavior, and interpretability were considered together in 2024 (RMSE = 0.047, MAE = 0.042, MAPE = 20.7%, sMAPE = 21.27%, MASE = 0.932). It accurately captured both seasonal and trend components of the infection rate series. While other models (ETS, Prophet, NNETAR) showed certain advantages, they were less satisfactory in terms of residual independence, normality, and forecasting stability.

These findings suggest that the SARIMA model is a robust and reliable tool for short-term MDROs infection forecasting, offering valuable support for antimicrobial stewardship, infection prevention strategies, and hospital resource allocation. Moreover, this study lays the groundwork for future research integrating multivariate or deep learning models to enhance predictive accuracy.

## Data Availability

The original contributions presented in the study are included in the article/Supplementary material, further inquiries can be directed to the corresponding author.
